# Health-related quality of life after TBI: a systematic review of study design, instruments, measurement properties, and outcome

**DOI:** 10.1186/s12963-015-0037-1

**Published:** 2015-02-17

**Authors:** Suzanne Polinder, Juanita A Haagsma, David van Klaveren, Ewout W Steyerberg, Ed F van Beeck

**Affiliations:** Erasmus MC, Department of Public Health, PO Box 2040, 3000 Rotterdam, CA The Netherlands

**Keywords:** Traumatic brain injury, Systematic review, Health-related quality of life, Functional outcome, Methodology

## Abstract

Measurement of health-related quality of life (HRQL) is essential to quantify the subjective burden of traumatic brain injury (TBI) in survivors. We performed a systematic review of HRQL studies in TBI to evaluate study design, instruments used, methodological quality, and outcome. Fifty-eight studies were included, showing large variation in HRQL instruments and assessment time points used. The Short Form-36 (SF-36) was most frequently used. A high prevalence of health problems during and after the first year of TBI was a common finding of the studies included. In the long term, patients with a TBI still showed large deficits from full recovery compared to population norms. Positive results for internal consistency and interpretability of the SF-36 were reported in validity studies. The Quality of Life after Brain Injury instrument (QOLIBRI), European Brain Injury Questionnaire (EBIQ), Child Health Questionnaire (CHQ), and the World Health Organization Quality of Life short version (WHOQOL-BREF) showed positive results, but evidence was limited. Meta-analysis of SF-36 showed that TBI outcome is heterogeneous, encompassing a broad spectrum of HRQL, with most problems reported in the physical, emotional, and social functioning domain. The use of SF-36 in combination with a TBI-specific instrument, i.e., QOLIBRI, seems promising. Consensus on preferred methodologies of HRQL measurement in TBI would facilitate comparability across studies, resulting in improved insights in recovery patterns and better estimates of the burden of TBI.

**Electronic supplementary material:**

The online version of this article (doi:10.1186/s12963-015-0037-1) contains supplementary material, which is available to authorized users.

## Introduction

It is important to obtain more insight in the measurement of health-related quality of life (HRQL) of patients with a traumatic brain injury (TBI), since there is a great need to document people’s pathways to recovery and to quantify the impact of TBI on population health over time. Although the mortality of TBI has decreased substantially in recent years, there has not been a proportionate reduction in disability due to TBI [1]. Disability is increasingly considered an important component of population health in general and more specifically of significance for the field of injury prevention and trauma care [2,3]. TBI is a leading cause of long-term impairments and disabilities in functional, physical, emotional, cognitive, and social domains [4,5]. Disability is a complex construct and can be measured using functional outcome scales or quality of life instruments. In the field of TBI outcome research, functional measurement scales are often used to assess disability after TBI [1]. Frequently used measures are the Glasgow Outcome Scale (GOS), GOS Extended (GOSe), Disability Rating Scale (DRS), Functional Independence Measure (FIM), Functional Assessment Measure (FAM), and the Functional Status Examination (FSE) [6]. Functional measurement scales are useful to portray functional problems but do not capture the patient’s subjective experience of their problems [6].

A more holistic and complete outcome measure is HRQL [1]. Quality of life is defined by the WHO as: “the individuals’ perception of their position in life in the context of the culture and value systems in which they live and in relation to their goals, expectations, standards and concerns. It is a broad-ranging concept affected in a complex way by the persons’ physical health, psychological state, level of independence, social relationships, personal beliefs and their relationship to salient features of their environment” [7]. From this definition, it becomes clear that the key factor in quality of life is the perception by the individual of his functioning. Since quality of life is a broad concept and may be influenced by numerous factors, the concept HRQL was developed. HRQL reflects an individual’s perception of how an illness and its treatment affect the physical, mental, and social aspects of his or her life [8]. These three domains (physical, mental, and social functioning) are, however, regularly assessed without evaluating the consequences of impairment on a patient’s life, so without a patient’s evaluation of his functioning. In these cases, only health status is measured. It may be evident that quality of life is often confused with the health status.

Whilst HRQL as an outcome measure in medicine has been used for over 30 years, it is only since the past decade that it is used in patients with a TBI [9]. Past perception was that TBI survivors would not be able to adequately rate their quality of life. As TBI encompasses multiple transient and permanent types of impairment, HRQL is recognized as an outcome variable that can provide well-standardized information on patient-perceived recovery after onset of the disease. A prerequisite to examining HRQL in patients with TBI is the availability of appropriate measures.

HRQL is usually assessed by questionnaires that will be filled out by the patient. Hence, more recently, these questions are referred to as patient-reported outcome measures (PROMs). HRQL instruments can be generic or disease-specific. Generic instruments do not take a particular condition into account and therefore allow comparisons with healthy individuals along with comparisons across various disease states. Disease-specific instruments take into account a patient’s specific health condition and therefore may be more sensitive to the consequences of the condition and more relevant to patients [6]. These instruments do allow comparisons with healthy individuals but not with patients with other diseases. A disease-specific HRQL measure for TBI, the Quality of Life after Brain Injury instrument (QOLIBRI) has been recently developed [10].

Some earlier reviews on the quality of life of patients with a TBI have been performed. Berger et al. [11] have discussed the literature published before 1999 on quality of life after TBI. They found 16 studies reporting at least two domains of quality of life. Five of these considered all four domains of quality of life (physical, psychological, social, and cognitive). However, most studies identified focused on the use of impairment scales and only one reported HRQL. Di Battista and colleagues [12] performed a review on quality of life in children and adolescents post-TBI. Eleven studies were included, of which seven used a HRQL measure. Furthermore, reviews on quality of life after TBI have been performed for specific subgroups, as mild TBI [13], and combat veterans [14]. In general it is stated that HRQL instruments have not yet been widely used in patients with a TBI [9,12,15] and that little is known about the HRQL of patients with TBI [9].

Particularly lacking is knowledge about the validity of HRQL instruments in patients with a TBI. Studies of high methodological quality are needed to guarantee appropriate conclusions on measurement properties (e.g. reliability, validity, and responsiveness).

This systematic review and quality assessment was conducted to describe the current state of knowledge in this field, with the aspiration to contribute to further consensus development on preferred methodologies for HRQL measurement within the TBI research field. We aimed: I) to evaluate the methodology of studies that purported to measure HRQL in patients with a TBI; II) to provide a narrative overview and perform a meta-analysis of HRQL of the most frequently used HRQL instrument(s) in patients with a TBI to gain insight into general recovery patterns and residual disability; and III) to evaluate the measurement properties of HRQL instruments used in patients with a TBI using the Consensus-Based Standards for the Selection of Health Status Measurement Instruments (COSMIN) checklist [16].

## Materials and methods

### Data sources and search strategy

Searches of eligible studies were conducted in Medline (PubMed), Web of Science, and Embase. All peer-reviewed articles published in the period January 1991 to July 1, 2013 were included in the searches. An electronic search strategy was developed in collaboration with a librarian who had extensive experience with systematic reviews. Search terms used were: “traumatic brain injury”, “brain injury”, “head injury”, “quality of life”, “health status”, “health status indicators”, “disability evaluation”, “functional outcome”, “activities of daily living”, “health status measure”, and “cohort studies” (see Annex I for search strategy). Keywords were matched to database-specific indexing terms. In addition to database searches, reference lists of review studies and articles included in the review were screened for titles that included key terms [17]. We searched for studies using HRQL measures and focused on traumatic brain injury as a consequence of a nondegenerative, noncongenital insult to the brain through an external mechanical force.

### Selection criteria

#### Inclusion criteria

A study had to meet the following criteria to be included in this review:

1. The target population had to be patients with a TBI suffering from any type and cause of trauma and any degree of severity (mild, moderate, severe);
2. Have generic or disease-specific HRQL as outcome measure;
3. The study had to be published in the period January 1991 to July 1, 2013;
4. The study had to be a randomized controlled trial, cohort study, case control study, clinical trial, or validation study of HRQL instruments;
5. The full abstract had to be available and the original peer-reviewed article published in English or German.

#### Exclusion criteria

1. Nontrauma-related TBI (e.g., tumor, hydrocephalus, general encephalopathy [includes bacterial and viral], stroke, birth-related trauma, genetic disorders affecting brain development and/or maturation [e.g., micro/macroencephalopathy, prematurity, agenesis of corpus callosum]) [12];
2. HRQL studies focusing on injury patients in general (including TBI as subgroup);
3. Studies concerning people other than the TBI patient.

### Data extraction

Relevant papers were selected by screening the titles (first step), abstracts (second step), and entire articles (third step), retrieved through the database searches. During each step, respectively, the title, abstract, or entire article was screened to ensure that it met the selection criteria listed above. This screening was conducted independently by two researchers (SP and JH). Disagreement between the reviewers about eligibility was resolved through discussion. Full articles were critically appraised by two reviewers (SP and JH), using data extraction forms. Their reports were compared and disagreements were resolved by discussion.

### Meta-analysis of the SF-36 in patients with a TBI

The SF-36 comprises eight HRQL domains: physical functioning, role limitations-physical, bodily pain, general health, vitality, social functioning, role limitations-emotional, and mental health. These domains can be aggregated into the physical component summary (PCS) and mental component summary (MCS) as weighted sums of the domain scores [18]. We collected the mean and standard deviations of the eight SF-36 health domains and the summary scores. If SF-36 scores had to be read from a graph, we rounded off to the nearest 0.5 points. If the study reported quality of life at multiple time-points, we chose the time-point closest to one year after TBI. We conducted random effects meta-analysis of study-specific mean SF-36 scores for the eight domains and two summary scores. We used the standard error of the mean score (the standard deviation of the score divided by the square root of the study size) for calculation of inverse-variance weights (I^2^) in the meta-analysis. Furthermore, we investigated the heterogeneity between studies by adjustment for study mean scores of SF-36 domains.

### Analysis of measurement properties of HRQL instruments in patients with a TBI

Based on the results from the data search strategy from the general review, we separately analyzed all studies that performed a quality assessment of a HRQL instruments in patients with a TBI. We used the Consensus-Based Standards for the Selection of Health Status Measurement Instruments (COSMIN) to evaluate the methodological quality of studies on measurement properties [16]. The checklist is developed for studies on health-related patient-oriented outcomes to rate the quality of the studies investigating psychometric qualities of measures. It can be used to assess whether an instrument meets the COSMIN standard for good methodological quality with regard to three quality domains, i.e., reliability, validity, and responsiveness, pertaining to one or more measurement properties. Reliability is defined as the extent to which scores for patients who have not changed are the same for repeated measurement under several conditions. Reliability contains the measurement properties internal consistency and reliability. Validity is the extent to which a questionnaire measures the construct it is intended to measure and contains content and construct validity (subdivided into structural validity and hypothesis testing). Responsiveness is the ability of an instrument to detect change over time. The domain responsiveness contains only one measurement property. Furthermore, interpretability is tested, which is the degree to which one can assign qualitative meaning to an instrument’s quantitative scores or change in scores [19]. The COSMIN checklist consists of nine boxes with five to 18 items concerning methodological standards for how each measurement property should be assessed. Each item was scored on a four-point rating scale (i.e., “poor”, “fair”, “good”, or “excellent”), which is an additional feature of the COSMIN checklist (see http://www.cosmin.nl) [20]. A methodological quality score per measurement property is obtained by taking the lowest rating of any of the items in a box. The methodological quality of a study was evaluated for each measurement property. Data extraction and assessment of (methodological) quality were performed by two reviewers (SP and JH) independently. Their reports were compared and disagreements were resolved by discussion.

## Results

### Literature search

**Figure 1:**
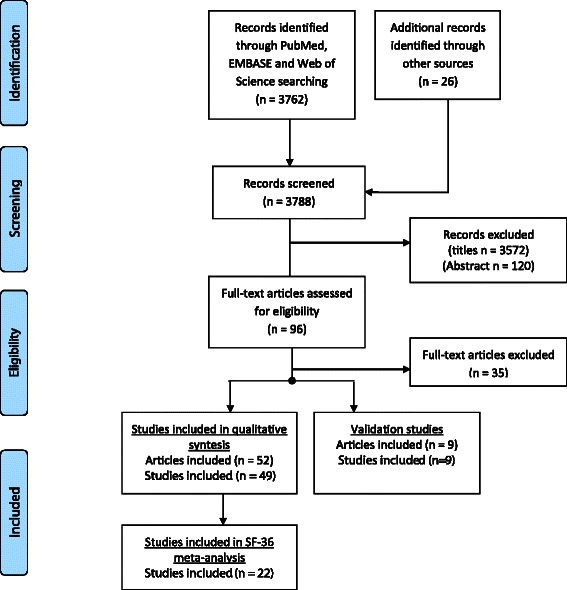
**Flow diagram of the search.**

The database search resulted in 3,762 unique titles of potentially relevant articles (Figure 1). Screening of the titles and abstracts resulted in a selection of 96 articles that appeared to meet all selection criteria. Thirty-five of these articles did not meet the inclusion criteria after the paper had been fully read, resulting in the final inclusion of 61 articles: 52 articles (describing 49 studies) measuring HRQL in patients with a TBI in general and nine studies with the main aim to validate a HRQL instrument in patients with a TBI. The main reasons for exclusion were not using a HRQL instrument or that the populations under study were injury patients in general (including TBI as subgroup).

### Study characteristics

Sample sizes of the studies varied widely, between 20 and 1,858 participants, with most studies having sample sizes below 200 participants (Table 1). Of the 49 studies included in our systematic review, 10 studies assessed HRQL in children only [21-31]. Not all studies reported demographics, but of those that did the study population of adult studies was above 14 years old (most studies applied an inclusion criterion of age 14 or 15+). None of the studies reported HRQL of both children and adults. In all studies reporting demographics, more males were included in the study (except one [32]). Not all studies reported a TBI severity level of their sample (n = 6, [26,31,33-36]). TBI severity was most often classified using the Glasgow Coma Scale (GCS, n = 36, Additional file 1: Table S1). The GCS scores can be translated into three levels: mild, moderate, and severe TBI. Twenty-one studies included all TBI severity levels (of which 17 used the GCS). A minority of studies only focused on severe (n = 3) or mild TBI (n = 8). Most of the studies included TBI patients with varying levels of severity (n = 20).

**Table 1:** **Study characteristics of 49 studies measuring HRQL in patients with a TBI**

Study characteristics	Studies (n)
**Number of HRQL instruments**	
1 instrument	39
2 instruments	7
> = 3 instruments	3
**Number of assessment time points**	
1 time point	30
2 time points	9
> = 3 time points	10
**Patient sample size**	
0-50	11
50-100	13
100-200	13
200-300	5
300-500	4
500+	3
**Patient population: age**	
Child studies	10
Adult studies (15+ years)	39
**Assessment time points**	
Pre-injury	5
Baseline	8
3-4 weeks	22
3 months	10
6 months	9
1 year	18
1-3 years	7
3-5 years	11
5-10 years	5
>10 years	5
**Proxy report** ^**1**^	
Yes	20
No	15
n.a.	14

^1^Whether a study used a proxy report instead of or besides patient reports.

### Measurement of HRQL

Eighteen different instruments were used to assess HRQL. Of the available instruments, the SF-36 was most often used (n = 29 (59%), Additional file 1: Table S1). The Sickness Impact Profile (SIP; n = 6), Pediatric Quality of Life Inventory (PedsQL; n = 5), EQ-5D, World Health Organization Quality of Life Instrument (WHOQOL(−BREF)) and Perceived Quality of Life Scale (PQOL; n = 3), and the QOLIBRI and Child Health Questionnaire (CHQ; n = 2) were used more than once. There were 10 HRQL instruments that were only used in one study. Nine studies used more than one HRQL instrument to measure HRQL (Table 1). Eight of these studies used the SF-36 (or SF-12) as well as another HRQL measure. When examining whether the choice of instrument depended on the severity level of the study population we found that the three studies only focusing on severe TBI all used a different measure (SARAH network Quality of Life questionnaire (SARAH), SIP, and SF-36), but that from the eight mild TBI studies, seven of them used the SF-36. In the 10 studies among children, nine used a HRQL instrument especially developed for children (and parents), and one study used an instrument that was not age-specific (15 Dimensions quality of life scale (15-D) [23]).

Assessment time points of quality of life varied widely and ranged from pre-injury to 24 years post-TBI. Seventeen studies (35%) used a longitudinal design with multiple assessments over time. HRQL was assessed most frequently at 1 month, one year, and 3 to 5 years following TBI (Table 1). Eight studies included “baseline assessments” conducted within 1 month after injury [22,24,26,31,32,37-40].

### Meta-analysis: SF-36 in patients with a TBI

**Figure 2:**
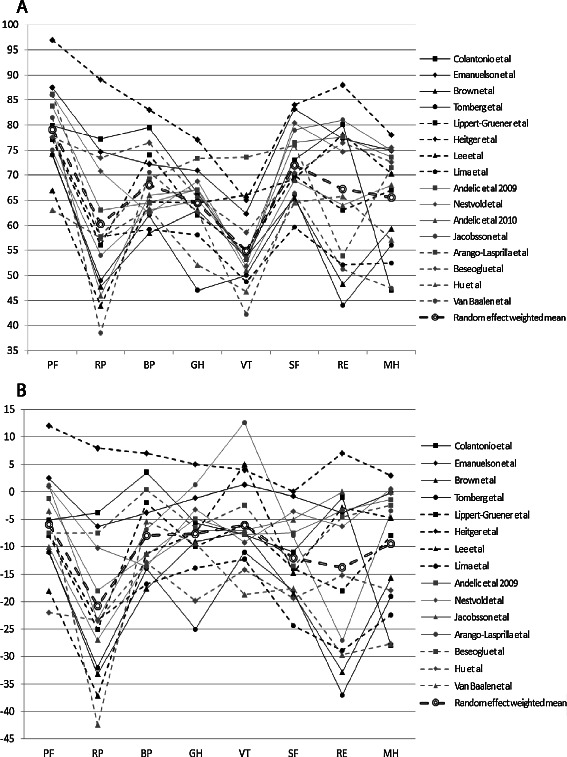
**SF-36 outcomes for eight dimensions for 17 studies. A**. SF-36 outcomes for eight dimensions and the random effect weighted mean. **B**. SF-36 outcomes for eight dimensions: difference with US norm scores. PF physical functioning, RP role limitation due to physical health, BP bodily pain, GH general health, VT vitality, SF social functioning, RE role limitation due to emotional problems, MH mental health.

**Figure 3:**
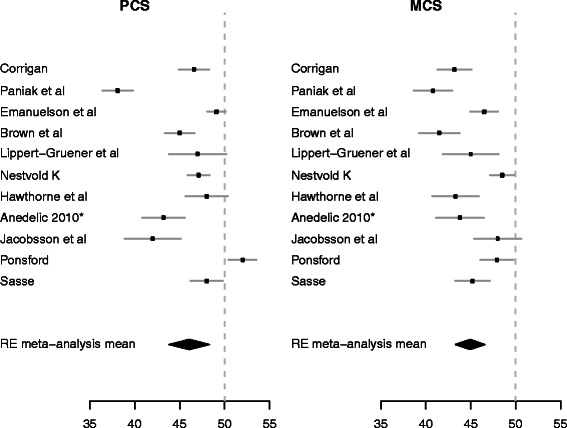
**Random effect meta-analysis of the SF-36 PCS and MCS for 11 studies.** MCS mental component summary score, PCS physical component summary score.

Of 29 studies that assessed HRQL with the SF-36, nine described all eight subdomains of the SF-36 plus the physical and mental component summary scores. Furthermore, 10 studies describe only the eight subdomains, and eight studies included only the mental and physical component scores. There were two studies that did not report outcomes, but only used SF-36 in multivariate regression models as a variable [34,41]. The standard deviations of the eight subdomains and two summary scores were reported by 13 and 11 studies, respectively. Twenty-two SF-36 studies were included in the meta-analysis. Random effects meta-analyses showed that the study-specific mean scores, although following the same patterns within these studies, were very heterogeneous beyond chance across these studies with I^2^ ranging from 0.83 to 0.97. Among the eight domains of the SF-36, role limitations due to physical health and vitality had lowest scores for patients with a TBI. Compared to the population norm scores, role limitations-physical and -emotional and social functioning had the lowest scores for patients with a TBI (Figure 2). Nine out of 15 studies that reported summary scores reported lower scores for MCS than PCS (Figure 3). The meta-analysis showed that studies also reported very heterogeneous results for the MCS and PCS (I^2^ 0.96 and 0.91, respectively). Adjustment for heterogeneity between studies did result in a slightly lower I^2^, but mainly showed that the residual heterogeneity is substantial.

### Measurement properties of HRQL instruments used in patients with a TBI

Nine studies, evaluating six different HRQL questionnaires, could be included in our quality assessment study [42-50]. The general characteristics of these studies are presented in Table 2. Three studies validated the SF-36 in patients with a TBI. Other instruments that were validated were the QOLIBRI, QOLIBRI-OS, EBIQ, CHQ, and the WHOQOL-BREF. The methodological quality of the studies is presented in Table 3 for each measurement property. Generally, the methodological quality of the studies per measurement property was fair to good. For five studies, at least half of the information regarding measurement properties is lacking [42,43,45,47,48]. The only study that measured reliability, validity, and responsiveness was the study by Chui et al. [49].

**Table 2:** **Study characteristics of nine validation studies of HRQL instruments in patients with a TBI**

First author, year, reference	HRQL instrument^1^	Country	Study population^2^	Assessment time points
Findler, 2001 [42]	SF-36	USA	n = 326 (M: 60%). RR: n.a. Age: 16–64 (mean: 34).	Variable: at least 1 year post-injury
MacKenzie, 2002 [43]	SF-36	USA	n = 1230 (M: 66%). RR: 78% Age: 18–59 (mean: n.a.)	1 year post-injury
Guilfoyle, 2010 [44]	SF-36	United Kingdom	n = 456 (M: 76%). RR: 88% Age: 18+ (mean: 37)	Between 1 and 24 months after TBI (mean 6 months)
Von Steinbuechel, 2010 [45]	QOLIBRI	Belgium, Finland, France, Italy, Netherlands, UK, USA, Australia, and Germany	n = 573 (M: 72%). RR: 62% Age: 15+ (mean: 39)	Between 3 months to 15 years post-injury (mean: 5 years)
Lin, 2013 [50]	QOLIBRI	Taiwan	N = 301 (M: 61%). RR: 97%% Age: 15+ (mean: 40)	During admission and 1 year
Von Steinbuechel, 2012 [46]	QOLIBRI-OS	Germany	n = 153 (M: 67%). RR: 62% Age: 15+ (mean: 39)	between 3 months to 15 years post-injury (mean: 5 years)
Teasdale, 1997 [47]	EBIQ	Belgium, Finland, France, Italy, Netherlands, UK, USA, Australia, and Germany	n = 258 (M: 62%). RR: n.a. Age: 16–93 (mean: 48)	Mean 31.8 months post- injury
Thomas-Stonell, 2006 [48]	CHQ	Canada	n = 33 (M: 67%). RR: n.a. Age: 4–18 (mean: 13)	During admission and follow-up - 11–150 days (mean: 38 days)
Chiu, 2006 [49]	WHOQOL-BREF	Taiwan	n = 199 (M: 64%). RR: 56% Age: (mean: 45)	Discharge (mean: n.a.)

^1^CHQ = Child Health Questionnaire; EBIQ = European Brain Injury Questionnaire; QOLIBRI = Quality of Life after Brain Injury; QOLIBRI-OS = QOLIBRI overall scale; SF-36 = Medical Outcome Study Short form-36 items; WHOQOL-BREF = Short version of the WHOQOL.

^2^Study population: N = sample size responders; M = males: RR = response rate.

**Table 3:** **Methodological quality per measurement property in nine validation studies**

Authors, year, reference	Instrument^1^	Internal consistency	Reliability	Content validity	Structural validity	Validity-Hypotheses testing	Responsiveness	Interpretability
Findler, 2001 [42]	SF-36	Fair				Good		
MacKenzie, 2002 [43]	SF-36	Good		Good		Good		
Guilfoyle, 2010 [44]	SF-36	Good	Excellent			Fair		Excellent
Von Steinbuechel, 2010 [45]	QOLIBRI			Excellent		Good		
Lin, 2013 [50]	QOLIBRI			Excellent		Good	Good	Good
Von Steinbuechel, 2012 [46]	QOLIBRI-OS	Excellent		Excellent	Excellent	Good		Fair
Teasdale, 1997 [47]	EBIQ	Excellent		Fair				
Thomas-Stonell, 2006 [48]	CHQ					Fair		
Chiu, 2006 [49]	WHOQOL-BREF	Excellent	Good	Good		Excellent	Good	Good

^1^CHQ = Child Health Questionnaire; EBIQ = European Brain Injury Questionnaire; QOLIBRI = Quality of Life after Brain Injury; QOLIBRI-OS = QOLIBRI overall scale; SF-36 = Medical Outcome Study Short form-36 items; WHOQOL-BREF = Short version of the WHOQOL.

We summarized the evidence of the SF-36 as measurement instrument based on three studies [42-44]. The SF-36 was designed to measure HRQL across eight domains. The three studies measuring validity of SF-36 in TBI were methodologically sound. There is moderate evidence for internal consistency for all SF-36 domains (Cronbach’s alpha = 0.68–0.92). Regarding interpretability, floor effects were observed in two domains, and ceiling effects were observed in four domains [44]. Differences in scores for subgroups (e.g., mild and severe TBI) could be detected. There was no information on structural validity and responsiveness.

For the QOLIBRI, EBIQ, and CHQ, only limited information is available based on one or two studies. The QOLIBRI demonstrated good internal consistency, content validity, and responsiveness [45,50]. The QOLIBRI-OS has good internal consistency and content and structural validity [46]. The WHOQOL-BREF showed good reliability, validity, and responsiveness [49].

## Discussion

This systematic review of HRQL measurement in patients with a TBI aimed to provide a comprehensive insight into the methodological quality of the papers, study design, and HRQL of patients with a TBI. There was considerable methodological variation between studies, including different instruments, study population (mix), follow-up periods, and timings of assessment. The SF-36 was the most widely used HRQL instrument to estimate quality of life of patients with TBI. The validity of the SF-36 was evaluated most frequently and showed reasonable internal consistency and interpretability. The meta-analysis of SF-36 studies showed that TBI is a heterogeneous condition that encompasses a broad spectrum of HRQL. Patients with a TBI particularly reported low scores for role limitations-physical and -emotional and social functioning.

Our review is one of the few studies that have considered the measurement of HRQL in TBI. Some earlier reviews on the quality of life of patients with a TBI have been performed. The literature review of Berger [11] published in 1999 identified 16 studies on quality of life, measured with a functional measurement instrument. Only one of these studies measured HRQL and was included in our review [51]. Recently, Di Battista and colleagues [12] performed a review on quality of life in children and adolescents post-TBI, in which 11 studies were found. Only five of these studies were included in our review [24-28]. The other six studies were not included, as they did not use a HRQL instrument (n = 5) or because they measured HRQL in caregivers [52]. In total, our review included 44 new studies assessing HRQL in patients with a TBI.

The review may be limited by the nature of the search strategies and corresponding target words used across databases. Although every attempt was made to ensure that articles relating to the construct of HRQL were included, it is possible that some articles were missed as a result of the breadth of database searches and the vast amount of literature the search yielded. To avoid this we used a variety of literature databases, and keywords were matched to database-specific indexing terms, although some studies might still have been missed. Furthermore, reference lists of review studies and articles included in the review were screened for titles that included key terms.

Decreased HRQL during and after the first year of TBI was a common finding of the studies included. TBI negatively impacts the mental HRQL (MCS) more strongly than the physical HRQL (PCS). In the long-term, patients with a TBI, on average, still showed large deficits from full recovery when measured by population norms. Among the eight domains of the SF-36, we found that role limitations-physical (RP) and -emotional (RE) had the lowest scores for patients with a TBI, compared to the population norm scores. It is notable that Guilfoyle et al. [44] found marked ceiling and floor effects for both the RP and RE domains. Presumably reflecting the limited number of possible scores in the RP and RE, they are inadequate for detecting change in patients with a TBI.

Our review reveals that there is still a lack of consensus about preferred HRQL instruments and study designs in the TBI field given the wide variety of different approaches used in the included studies. It is remarkable that in the 49 papers reviewed, 18 different HRQL instruments were used. Decisions regarding which HRQL measure to use will be influenced by a range of factors, e.g., availability in own language, availability of normative population values, user fees, and instrument length. Different HRQL instruments assess different domains of health, which make the comparison of study outcomes difficult. Variations in descriptive systems, weightings, and scale ranges between HRQL instruments result in different outcomes (utilities) for similar health states [53,54]. This supports the need for guidelines for the conduct of follow-up studies measuring TBI-related HRQL. Before guidelines can be developed for the measurement of HRQL in TBI, several general methodological issues that arise from the incorporation of HRQL measurement into research need to be addressed. Examples of these issues include HRQL instrument selection (Which one? How many?); timing of HRQL measurements (How often? Over what period of time?); and interpretation of results (medical outcomes versus HRQL outcomes, proxy versus self-report) [9].

### Challenges in HRQL assessments in TBI

The assessment of general HRQL in patients with severe TBI, cognitive impairments, and/or very young age is difficult. HRQL measures should be used with caution in these patient groups [6,55]. More research is needed into how HRQL measures could be modified to make them more suitable for people with severe brain damage and/or cognitive impairments.

Another major question is whether and under which conditions patients can self-report on their HRQL. Furthermore, in patients who cannot self-report on their HRQL, proxy ratings can be helpful, however taking into account that if the patient could respond, his or her judgment could differ significantly from that of the proxy. Whether a proxy can fill in a questionnaire on someone’s “subjective experience” is under discussion. Self- and proxy-report should thus be considered as complementary sources of information and not as equal replacements [9].

### Generic versus disease-specific instruments

For the TBI research field it is important to make a choice about which HRQL instrument or combination of instruments (generic and disease-specific) can best be used in patients with a TBI. There has been extensive discussion about the advantages and disadvantages of generic versus disease-specific HRQL measures. Berger et al. [11] claim that generic HRQL measures are not appropriate for research into TBI and its treatment because the measures do not cover domains that are typically significantly affected—especially cognitive functioning. A similar criticism is leveled by Bullinger et al. [56] who also specified that existing instruments do not cover the “existential domain”. Including that domain would expand the coverage of HRQL measures into the area typically encompassed by “social well-being”. Riemsma et al. [55] questioned the validity and applicability of most generic quality of life measures to TBI because of self-report problems and the inadequate coverage of salient domains. Von Steinbuechel [10] states “that the development of a disease-specific HRQL measure for TBI opens the possibility of constructing a composite outcome assessment that covers both functional outcomes and HRQL”. Such a composite assessment would help to complete the picture of outcome after brain injury and potentially give a more sensitive assessment for clinical trials. In contrast, Dijkers [57] questioned the need to develop disease-specific TBI HRQL measures and suggests that it may be more efficient to explore whether it is possible to develop modules that quantify quality of life in areas typically omitted in generic quality of life measures—for example, cognitive functioning.

We found that the SF-36, a generic instrument, is currently the most widely used instrument to assess HRQoL after TBI. It’s available in several languages, has availability of population norms for many countries, and its track record in other disorders may be the underlying cause of the extensive use of the SF-36 in TBI [6].

An advantage of the SF-36 is that it can be used to estimate a preference-based single-index measure for health using general population values (and therefore, it can be used in economic evaluations). The validity of the SF-36 was evaluated in three studies and showed positive results for internal consistency and interpretability. However, the SF-36 has some limitations in its application in TBI patients. The SF-36 may not be sensitive enough to detect key issues in patients with a TBI, such as cognitive dysfunction or severe physical restrictions, or patients with psychological problems, such as anxiety, depression, memory, and concentration disturbances. Furthermore, floor and ceiling effects should be kept in mind if selecting individual domains as outcome measures, particularly in the context of clinical trials, since reduced responsiveness to change increases the risk of not detecting a clinically important treatment effect [44]. According to the current evidence, role limitations due to physical health and emotional problems (RP and RE) are less suitable as outcome measures, since marked ceiling and floor effects were found for both the RP and RE domains. Furthermore, Findler et al. [42] noted that the SF-36 may be a more sensitive measure of health-related problems in patients with mild TBI than in those with moderate and severe TBI, since the correlations between the SF-36 domains and measures of health problems associated with TBI were weaker and more uniform in the moderate and severe TBI group (compared to the correlations in the mild TBI group).

The recently developed TBI-specific HRQL instrument the QOLIBRI seems promising. An international validation study has recently been published showing that the QOLIBRI is a valid instrument in a TBI population, has good correlation with the GOSe, and provides additional information to that obtained by the SF-36 [45]. The validation study did not assess the QOLIBRI’s ability to detect change in HRQL over time (that is, its responsiveness). The QOLIBRI is validated in German, Finnish, Italian, French, English, and Dutch [58]. It does not yet appear to have been used outside of validation studies, and further research is required to determine its responsiveness [6].

### The future of HRQL measurement in TBI

In 2002, an international group of clinicians and quality of life experts made several recommendations for future research of quality of life in patients with a TBI [56]. With regard to the measurement time point, HRQL assessment was recommended to take place not upon admission to the hospital, but in the early phase of rehabilitation (i.e., within 1 year after trauma) and in the post-rehabilitation phase. Self-report should be preferred to proxy-report. Furthermore, the expert group recommended that the assessment of HRQL includes both a generic and condition-specific instrument [56]. An instrument or combination of instruments including key problem areas in patients with a TBI is needed to assess the full impact of TBI on HRQL. Therefore, we recommend using a generic measure (SF-36) with a disease specific measure (QOLIBRI).

Fundamental research (as input for guideline development) should be undertaken alongside work on the development and validation of specific instruments. In 1999 the National Institutes of Health (NIH) consensus conference on rehabilitation of persons with TBI made a broad recommendation that generic HRQL assessment instruments must be validated for use with persons with TBI and TBI-specific instruments [57]. To properly assess the measurement properties of HRQL instruments, studies of high methodological quality are needed. In our study we evaluated the validation studies with the COSMIN checklist [16]. The COSMIN checklist facilitates a separate judgment of the methodological quality of the included studies and their results. This is in line with the methodology of systematic reviews of clinical trials [59]. The studies included in this review used 18 different instruments to assess HRQL. Only six of these instruments have been validated in patients with a TBI. Validity assessment of HRQL measurements for people with TBI should be addressed in studies specifically designed for this patient population and should include information on reliability, validity, and responsiveness. When there is need for proxy assessments (e.g., in severe TBI, cognitive impairment, and children) the instrument should also be assessed for patient–proxy agreement and inter-rater agreement.

## Conclusions

In conclusion, this review shows that there is considerable variation in study design between studies measuring HRQL in TBI. There are still major gaps in our understanding of how to measure the impact of TBI on personal and population health. The use of the SF-36 in combination with a TBI-specific instrument, e.g., QOLIBRI, seems promising. Development of guidelines for the measurement of HRQL in TBI with validated instruments would facilitate comparability across studies, which would produce improved estimates of TBI disability and recovery patterns.

## ANNEX I: Search strategy Pubmed

(("brain injuries"[MeSH Terms] OR ("brain"[All Fields] AND "injuries"[All Fields]) OR "brain injuries"[All Fields] OR ("traumatic"[All Fields] AND "brain"[All Fields] AND "injury"[All Fields]) OR "traumatic brain injury"[All Fields]) OR ("brain injuries"[MeSH Terms] OR ("brain"[All Fields] AND "injuries"[All Fields]) OR "brain injuries"[All Fields] OR ("brain"[All Fields] AND "injury"[All Fields]) OR "brain injury"[All Fields]) OR ("craniocerebral trauma"[MeSH Terms] OR ("craniocerebral"[All Fields] AND "trauma"[All Fields]) OR "craniocerebral trauma"[All Fields] OR ("head"[All Fields] AND "injury"[All Fields]) OR "head injury"[All Fields])) AND (("quality of life"[MeSH Terms] OR ("quality"[All Fields] AND "life"[All Fields]) OR "quality of life"[All Fields]) OR ("activities of daily living"[MeSH Terms] OR ("activities"[All Fields] AND "daily"[All Fields] AND "living"[All Fields]) OR "activities of daily living"[All Fields]) OR ("health status"[MeSH Terms] OR ("health"[All Fields] AND "status"[All Fields]) OR "health status"[All Fields])) AND (1991[MHDA]: 2013[MHDA]).

## Additional file

Additional file 1: Table S1. Study characteristics of 49 studies measuring HRQL in patients with a TBI (in order of year/alphabetic; bold author names are studies of children) [5,21-41,51,53,60-87].
