# Supplementary material for: Health-related quality of life after TBI: a systematic review of study design, instruments, measurement properties, and outcome
Source: Popul Health Metr. 2015 Feb 17;13:4. doi: 10.1186/s12963-015-0037-1 (PMC4342191; doi:10.1186/s12963-015-0037-1)
Supplement: Additional file 1: Table S1. — Study characteristics of 49 studies measuring HRQL in patients with a TBI (in order of year/alphabetic; bold author names are studies of children) [5,21-41,51,53,60-87]. [file 12963_2015_37_MOESM1_ESM.doc]

**Additional file 1: Table S1.** Study characteristics of 49 studies measuring HRQL in patients with a TBI (in order of year/alphabetic; bold author names are studies of children)

| **First author, year, country, reference** | **Study population^1^** | **TBI severity (% mild, moderate, severe)^2^** | **HRQL instrument (mode of administration)^3^** | **Assessment time points^4^** | **Proxy^5^** |
| --- | --- | --- | --- | --- | --- |
| McLean, 1993, USA | n=102 (M: n.a.). RR: n.a. Age: 15-60 (mean: 26 y) | GCS: 58, 11, 28 | SIP | 1 and 12 months | n.a. |
| van Balen, 1996, Netherlands | n=51 (M: 55%). RR: 84%. Age: 15-30 (mean: 26) | GCS: 100% severe | SIP | 3-7 year | Yes |
| Corrigan, 1998, USA | n=95 (M: 70%). RR: 24%. Age: 14-74 (mean: 32) | n.a. | SIP, SF-36 | 6-60 months after inpatient rehabilitation | Yes |
| Colantonio, 1998, Canada | n=51 (M: 73%). RR: 88%. Age: 15-19 (mean: 18) | GCS: 47, 14, 39 | SF-36 | 5 years | Yes |
| Paniak, 1999, Canada | n=120 (M: 45%). RR: 100%. Age: (mean: 33) | Mild: 100% | SF-36 | Within 3 weeks | No |
| Wilson, 2000, United Kingdom | N=135 (M: 84%).RR: 100%. Age: 16-69 (mean 37) | GOSE: 39, 33, 29 | SF-36 | 5-10 months | Yes |
| Steadman, 2001, Canada | n=275 (M: 70%). RR: 71%. Age: 14+ (mean: 29) | n.a. | SF-36 | 8 -24 years | Yes |
| Dikmen, 2001 & 2003, USA | n=210 (M: 82%). RR: 80%. Age: 14+ (mean: 36) | GCS: 61, 22, 18 | SF-36, PQOL | 3-5 years | n.a. |
| **Stancin, 2002, USA** | n=84 (M: 73%). RR: 77%. Age: 6-12 (mean: 9) | GCS: moderate 50%, severe 50% | CHQ, Parent Form ([PF50, 50 items) and Child Form (CF87, 87 items) | Pre-injury, 3 weeks, 6 and 12 months, 4 years. | Yes |
| Emanuelson, 2003, Sweden | n=173 (M: 65%). RR: 35%. Age: 16-60 (mean: 32) | GCS: Mild 100% | SF-36 | 3 months and 1 year | n.a. |
| Brown, 2004, USA | n=135 (M: 73%). RR: 34%. Age: 16+ (mean: 34) | n.a. | SF-36 | 3 months | n.a. |
| Bell, 2005, USA | n=157 (M: 77%). RR: 92%. Age: 18-70 (mean: 36) | GCS: Severe 36%, mild/ moderate 64% | SF-36, EQ-5D, PQoL | 1 year | n.a. |
| **Horneman, 2005, Sweden** | n=109 (M: n.a.). RR: 67%. Age: <18 (mean: 13) | GCS mean 7.7 | 15-D | 10 years | n.a. |
| Tomberg, 2005, Estonia | n=85 (M: 81%). RR: 68%. Age: 14-66 (mean: 38) | GCS: Moderate 67%, severe 24% | SF-36 | average 2.3 years (range 9-36 months) | n.a. |
| Teasdale, 2005, Denmark | n=240 (M: n.a.). RR: 76%. Age: >15 (mean: 31) | GCS: 41, 13, 46 | EBIQ | 5, 10 or 15 years | n.a. |
| Van Baalen, 2006, the Netherlands | n=25 (M: 68%). RR: 88%. Age: 18-50 (mean: 35) | GCS: moderate to severe 100% | SF-36, SIP | 1 year post-injury | No |
| **McCarthy, 2006, USA** | n=330 (M: 69%). RR: n.a. Age: 5-15 (mean: n.a.) | AIS: mild (2-3) 56%, mod (4) 31%, sev (5) 13% | PedsQL | Baseline, 3 and 12 months | yes |
| McCarthy, 2006, USA | n=1858 (M: 64%). RR: 52% Age: 14+ (mean: 43) | AIS: mild (2) 33%, moderate to severe (>2) 67% | SF-36 | 1 year | No |
| Pagulayan, 2006, USA | n=133 (M: 81%). RR: 27% Age: 14+ (mean: 37) | GCS: 65, 20, 7 (unknown 9%) | SIP | 1, 6, 12 months, and 3-5 year | n.a. |
| Svendsen, 2006, Denmark | n=37 (M: 70%). RR: 62%. Age: (mean: 26) | GOS: severe 3%, moderate /severe GCS: 28%, moderate 53%, moderate/good 16% | EBIQ, WHOQoL | 15-17 years | yes |
| Jakola, 2007, Norway | n=28 (M: 68%). RR: 30%. Age: 15-80 (mean: 32) | GCS: mild 100% | EQ-5D | 5-7 years | n.a. |
| Klose, 2007, Denmark | n=104 (M: 75%). RR: n.a. Age: 18-64 (mean: 41). | GCS: 44, 19, 38 | NHP, EQ-5D | Pre-injury, 3 and 12 months | yes |
| Lippert-Gruener, 2007, Germany | n=49 (M: 78%). RR: n.a. Age: 15-68 (mean: 32) | GCS: Severe 100% | SF-36 | 6 and 12 months | No |
| Heitger, 2007, New Zealand | n=37 (M: 65%). RR: n.a. Age: 15-65 (mean: 29) | GCS: mild: 100% | SF-36 | 1 week, 3, 6 and 12 months | No |
| **Souza, 2007, Brazil** | n=23 (M: 57%). RR: n.a. Age: 7-13 (mean: 11). | GCS: Severe 100% | SARAH for children and adolescents | average 4 years | Yes |
| **Petersen, 2008, Germany** | n=59 (M: n.a.). RR: n.a. Age: 4-20 (mean: n.a.) | n.a. | KINDL parent and child form | first contact moment and 3 months | Yes |
| Lee, 2008, Taiwan | n=114 (M: n.a.). RR: 92%. Age: 18+ (mean: n.a.) | n.a. | SF-36 | 3 months after hospital discharge | n.a. |
| Lima, 2008, Brazil | n=39 (M: 51%). RR: 78%. Age: (mean: 39) | GCS: Mild 100% | SF-36 | 18 months | Yes |
| Andelic, 2009, Norway | n=60 (M: 76%). RR: 97%. Age: 16-55 (mean: 41). | GCS: moderate 52%, severe 48% | SF-36 | 10 years | No |
| Nestvold, 2009, Norway | n=259 (M: 58%). RR: 72%. Age: n.a. (mean: 45) | mild (PTA<24 hours) 91% | SF-36, GHQ-30 | mean 22 years | No |
| Hawthorne, 2009, Australia | n=66 (M: 71%). RR: 61%. Age: >14 (mean: 39). | GCS: 48, 9, 43 | SF-36, AQoL, SF6D | Between 3 months - 15 years (median: 32 months) | No |
| **Limond, 2009, UK** | n=47 (M: n.a.). RR: 33%. Age: 5-16 (mean: 10) | GCS: 72, 16, 12 | PedsQL | 1-5 years | yes |
| Andelic , 2010, Norway | n=85 (M: 76%). RR: 58%. Age: 16-55 (mean: 31) | GCS: mean 7.8 | SF-36 | 1 year | No |
| Truelle, 2010, Belgium, Finland, France, Italy, Netherlands, UK, USA, Australia and Germany | n=573 (M: 72%). RR: 62%. Age: >15 (mean: 39) | GCS: 32, 10, 58 | QOLIBRI, SF-36 | between 3 months to 15 years (mean: 5 years) | No |
| Lin, 2010, Taiwan | n=158 (M: 55%). RR: 78%. Age: >15 (mean: 51) | GCS: 74, 18, 8 | WHOQOL-BREF | Discharge, 6 and 12 months | No |
| Jacobsson, 2010, Sweden | n=67 (M: 76%). RR: 63%. Age: 18-65 (mean: 39) | GCS: mild 48, mod/sev: 52 | SF-36 | 6-15 years | Yes |
| **Erickson, 2010, USA** | n=20 (M: 65%). RR: n.a. Age: 9-18 (mean: 13) | GCS: moderate/ severe 100% | PedsQL | > 6 months (mean : 4 years) | yes |
| Bell, 2011, USA | n=343 (M: 75%). RR: 79%. Age: 16+ (mean: 39) | GCS: 35, 17, 33, paralyzed 16% | SF-12, EQ-5D, PQoL | 1 and 2 year | n.a. |
| Ponsford, 2011, Australia | n=123 (M: 74%). RR: 63%. Age: 18+ (mean: 35) | GCS: mild 100% | SF-36 | Pre-injury, 1 week, 3 months | No |
| **Rivara, 2011 & 2012 & 2013, USA** | n=769 (M: 66%). RR: 62%. Age: <18 (mean: 9) | GCS: 85, 13, 2 | PedsQL | Pre-injury, 3, 12, 24, and 36 months | Yes |
| **Anderson , 2012, Australia** | n=205 (M: 70%). RR: 40%. Age: 6-14 (mean: 11) | GCS: 63, 27, 10 | CHQ | Pre-injury, 6 months | Yes |
| **Swanson, 2012, USA** | n=347 (M: 71%). RR: n.a. Age: 2-17 (mean: n.a.). | n.a. | PedsQL | Baseline and 12 months | Yes |
| Arango-Lasprilla, 2012, Colombia | n=31 (M: 61%). RR: 62%. Age: 18-65 (mean: 34) | GCS: moderate 90%, severe 10% | SF-36 | > 6 months (mean: 17 months) | No |
| Sasse, 2012, Germany | n=141 (M: 71%). RR: 41%. Age: 17-68 (mean: 45) | GCS: 31, 21, 23, complicated mild 18% | SF-36, QOLIBRI | Between 3 months and 15 years (mean: 40 months) | Yes |
| Hu, 2012, China | n=381 (M: 72%). RR: 84%. Age: 18+ (mean: 33) | GCS: moderate 59%, severe 41% | SF-36 | discharge, 6, 12, and 24 months after discharge | n.a. |
| Forslund, 2013, Norway | N=91 (M: 77%). RR: 68%.  Age= 16-55 (mean:31) | GCS: moderate 35%, severe 65% | SF-36 | 1 and 2 years | No |
| Beseoglu, 2013, Germany | n=36 (M: 67%). RR: n.a. Age: (mean: 51) | GCS: mild 100% | SF-36 | 3 years | n.a. |
| Maestas, 2013, USA | N=187 (M: 76%). RR: 83%. Age: (mean: 33) | GCS: mild 100% | SF-36 | 3 months | No |
| Williamson, 2013, USA | N=131 (M: 68%). RR: 22%. Age: (mean: 35) | AIS: 76% moderate, 24% severe | SIP | 2 years | No |

^1^ Study population: N=sample size responders; M= males: RR= response rate.

^2^ GCS scores were coded into three levels: mild (13–15), moderate (9–12), and severe (GCS score 3–8). Percentages are states as mild, moderate, severe GCS if not stated otherwise. AIS = Abbreviated Injury Scale.

**^3^** AQoL = Assessment of Quality of Life instrument; CHQ= Child Health Questionnaire; EBIQ = European Brain Injury Questionnaire; EQ-5D= European Quality of life instrument-5 dimensions; KINDL = Questionnaire for Measuring Health-Related Quality of Life in Children and Adolescents (German language); GHQ-30 = General Health Questionnaire 30 items; NHP = Nottingham Health Profile; PedsQL = Pediatric Quality of Life Inventory; PQOL = Perceived Quality of Life Scale; QOLIBRI = Quality of Life after Brain Injury; SARAH = SARAH network Quality of Life questionnaire; SF6D = Medical Outcome Study Short Form-6 dimensions; SF-12= Medical Outcome Study Short Form-12 items; SF-36= Medical Outcome Study Short form-36 items; SIP = Sickness Impact Profile; WHOQOL = World Health Organization Quality of Life; WHOQOL-BREF = Short version of the WHOQOL; 15-D = 15 Dimensions quality of life scale.

^4^ If not stated otherwise, the assessment time points are post-injury.

^5^ Whether a study used a proxy report instead of or besides a patient report.
